# Histopathological diagnosis of myocarditis in a dengue outbreak in Sri Lanka, 2009

**DOI:** 10.1186/1756-0500-4-268

**Published:** 2011-07-29

**Authors:** Kosala GAD Weerakoon, Senanayake AM Kularatne, Deepthika H Edussuriya, Sarachchandra KA Kodikara, Laxman PG Gunatilake, Vasanti G Pinto, Ashoka B Seneviratne, Sunethra Gunasena

**Affiliations:** 1 Department of Medicine, Faculty of Medicine, University of Peradeniya, Sri Lanka; 2 Department of Forensic Medicine, Faculty of Medicine, University of Peradeniya, Sri Lanka; 3 Department of Anaesthesiology, Faculty of Medicine, University of Peradeniya, Sri Lanka; 4 General Hospital, Kandy, Sri Lanka; 5 Department of Virology, Medical Research Institute, Colombo 8, Sri Lanka

## Abstract

**Background:**

In 2009, an outbreak of dengue caused high fatality in Sri Lanka. We conducted 5 autopsies of clinically suspected myocarditis cases at the General Hospital, Peradeniya to describe the histopathology of the heart and other organs.

**Methods:**

The diagnosis of dengue was confirmed with specific IgM and IgG ELISA, HAI and RT-PCR techniques. The histology was done in tissue sections stained with hematoxylin and eosin.

**Results:**

Of the 319 cases of dengue fever, 166(52%) had severe infection. Of them, 149 patients (90%) had secondary dengue infection and in 5 patients, DEN-1 was identified as the causative serotype. The clinical diagnosis of myocarditis was considered in 45(27%) patients. The autopsies were done in 5 patients who succumbed to shock (3 females and 2 males) aged 13- 31 years. All had pleural effusions, ascites, bleeding patches in tissue planes and histological evidence of myocarditis. The main histological findings of the heart were interstitial oedema with inflammatory cell infiltration and necrosis of myocardial fibers. One patient had pericarditis. The concurrent pulmonary abnormalities were septal congestion, pulmonary haemorrhage and diffuse alveolar damage; one case showed massive necrosis of liver.

**Conclusions:**

The histology supports occurrence of myocarditis in dengue infection.

## Background

Dengue virus infection is widely distributed in the tropical and subtropical regions of the globe affecting up to 100 million people per year; 2.5 billion people are at risk [1]. The infection is caused by 1 of 4 antigenically distinct but related single stranded RNA viruses in the family Flaviviridae and is transmitted by mosquito vectors, primarily *Aedes aegypti *[1]. Dengue virus infections cause a spectrum of illnesses ranging from self-limiting fever to severe dengue haemorrhagic fever(DHF) where increased vascular permeability is the main pathology leading to shock [1-3]. In some cases, uncommon complications such as acute hepatic failure, acute renal failure, dengue encephalitis and myocarditis have been recognized [4-7]. In general, many viruses cause myocarditis and their pathogenesis has been described in the literature. However, the dengue virus as a cause of viral myocarditis is not emphasized in most of these reports [8-14].

In Sri Lanka, dengue fever emerged in the early 1980s and all dengue viral serotypes, DEN-1, DEN-2, DEN-3, and DEN-4 have been found in circulation causing DHF [2,3] The worst recorded outbreak of dengue in the island occurred in 2009 with 24629 cases and 245 deaths reported [15]. During this epidemic, the General Hospital, Peradeniya in the hilly Central Province of the island received a substantial number of dengue cases. Of them, a proportion of patients died with predominant cardiac dysfunction, despite meticulous fluid management in the Intensive Care Unit. Cardiac complications in dengue fever were reported from the same unit in 2005, where 75 patients had ECG changes with cardiac dysfunctions, all patients recovering [4]. However, lack of histological diagnosis from myocardial biopsies was the main concern in this study to confirm dengue virus as a cause of myocarditis [4]. Therefore, in 2009, we performed a limited number of autopsies on patients who died due to dengue complications and the tissue material was subjected to histopathological examination. Material from five cases showed histopathological changes in the myocardium. In this study we aim to describe the histopathological features of the myocardium in these 5 cases and also to describe other organ system involvement and the main clinical manifestations they showed during the illness.

## Methods

### Settings and subjects

The Ethical Review Committee of the Faculty of Medicine, University of Peradeniya, Sri Lanka, granted ethics approval for this study, which was then carried out in compliance with the Helsinki Declaration. We studied a cohort of 319 adult dengue patients admitted to the Professorial Medical Unit, Teaching Hospital, Peradeniya from May to August 2009. This hospital is a tertiary care institution which receives patients transferred from the hospitals in the region. After obtaining informed written consent from each patient, clinical information including signs, symptoms and medical management were recorded in a standard data sheet. All the patients were managed according to the routine protocols of the unit. Investigations such as full blood count, haematocrit and liver biochemical tests were done in all patients whilst ultra sound scan of the abdomen, chest radiograph, ECG and 2D echocardiogram were done in severely ill patients to identify complications. The presence of significant ECG changes such as T wave inversion in many leads, ST segment depression, bundle branch blocks and arrhythmias, which reverted to normal after recovery were taken as criteria for the diagnosis of myocarditis. Positive troponin T test, hypokinetic wall motion and decreased ejection fraction in 2D echocardiogram were taken as markers of severe myocarditis.^4 ^The patients were categorised as having severe illness based on the WHO guideline-1997 with addendums, the criteria being fall of platelet count below 100 × 10^9^/l, significant spontaneous bleeding, evidence of third space fluid accumulation, and any vital organ dysfunction such as myocarditis, hepatic or renal involvement.

### Confirmation of the diagnosis

The disease was confirmed using dengue specific IgM and IgG, Haemagglutination Inhibiting Antibodies (HIA) test and viral identification with reverse transcriptase polymerase chain reaction agarose gel electrophoresis (RT-PCR-AGE) done at the Medical Research Institute, Colombo, Sri Lanka. RT-PCR-AGE was done for patients who presented within 4 days of onset of fever. The sera were tested by the HIA and for IgM antibody using IgM antibody capture ELISA. If only dengue virus specific IgM antibodies were detectable in the test sample, the patient was considered to have primary dengue infection, where as the presence of both IgM and IgG or HIA titre above 1: 2560, or both together, was considered as marking a secondary dengue infection. Blood samples for serology were collected on 5-7^th ^day of illness.

### Histopathological study

The autopsies were done by senior medical officers well experienced in autopsy studies with the participation of a member of the medical team. The macroscopic appearance of all organs was documented including dissected surfaces. The tissues samples were harvested from the heart and other organs, especially from macroscopically abnormal sites, and sent to the Department of Forensic Medicine, Faculty of Medicine, Peradeniya University for histopathological examination. Formalin fixed tissues were processed, embedded in paraffin, and sectioned. The sections were stained with hematoxylin and eosin for histological examination.

## Results

### Data of the cohort

**Figure 1:**
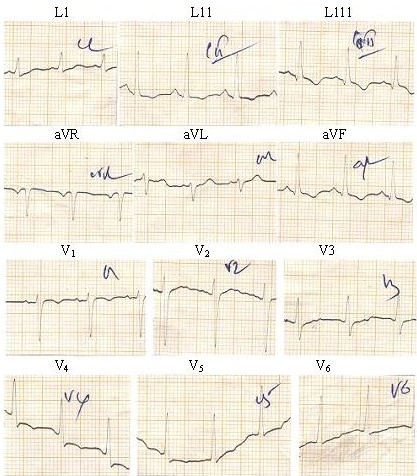
**An ECG showing wide spread T wave inversion in a 22-year-old female patient with dengue infection**.

Of the 319 patients, 166(52%) had severe infection with complications and most of them had more than one complication occurring simultaneously (table 1). Of them, 149 (90%) patients had secondary dengue infection. In 5 patients, DEN-1 was identified as the causative serotype among 22 blood samples tested with RT-PCR-AGE. These 22 patients were sero positive for secondary dengue infection. Severe thrombocytopenia manifested in most of the patients and 94 patients (57%) had dropping of platelet count below 50 × 10^9^/l. Forty five (27%) patients had clinical and ECG evidence suggestive of myocarditis (Figure 1) and of them, 21(13%) had concurrent pleural effusions. During the period of study 11 patients died in the ICU.

**Table 1:** Frequency of complications in 166 patients with severe dengue infection, 2009

Complication	No. of patients (%)
Thrombocytopenia (< 50 × 10^9^/l)	94 (57)
Significant spontaneous bleeding#	47 (28)
Hypotension (SBP < 90 mmHg)*	56 (34)
Provisional diagnosis of myocarditis	45 (27)
Pleural effusion	41 (25)
Myocarditis + pleural effusion	21 (13)
Ascites	29 (18)
Acute renal failure	06 (4)
Acute liver failure	34 (20)

*Systolic blood pressure, # persistent bleeding from skin or mucosa such as haematemesis or melena

### Clinical data of patients with histopathological evidence of myocarditis (table 2)

**Table 2:** Important histopathological findings of tissues obtained at autopsy and specific clinical data

Age (y) Gender	Major clinical manifestations	Histological findings
16,F	Effusions Hypotension Tachycardia ECG- T ↓ Troponin T + 2D echo +	Heart - Interstitial oedema, infiltration of acute inflammatory cells, necrosis of myocardial fibers (appearance of myocarditis). Lungs - capillary congestion, alveoli filled with histiocytes and fibrinous debris (appearance of diffuse alveolar damage).
28,M	Effusions Hypotension Tachycardia Spontaneous bleeding ECG- T ↓ Troponin T + 2D echo +	Heart - interstitial oedema, haemorrhage with scattered inflammatory cells and necrotic myocardial fibers, infiltration of pericardium by acute inflammatory cells (appearance of myocarditis and pericarditis). Lungs - extensive pulmonary oedema and haemorrhage. Brain - vascular congestion with cerebral oedema. Skeletal muscle - haemorrhage in perimycium.
28,M	Effusions Hypotension Tachycardia ECG- T ↓ Troponin T +	Heart - interstitial oedema, inflammation and necrotic muscle fibers (appearance of myocarditis). Lungs - septal congestion and haemorrhage with pulmonary oedema. Brain - meningeal congestion. Spleen - widened red pulp and haemorrhage.
31,F	Effusions Hypotension Tachycardia ECG- T ↓ Troponin T + 2D echo +	Heart - interstitial oedema, scattered necrosis of myocardial fibers surrounded predominantly by lymphocytes (appearance of myocarditis). Lungs - extensive neutrophilic exudates in alveoli with capillary congestion (appearance of severe pneumonia leading to acute respiratory distress syndrome). Liver - massive necrosis.
13,F	Effusions Hypotension Tachycardia Bleeding ECG- T ↓ Troponin T +	Heart - interstitial oedema and scattered necrosis of myocardial fibers surrounded predominantly by lymphocytes (appearance of myocarditis). Liver - Macrovesicular steatosis.

The series comprised 3 females and 2 males with their age ranging from 13 to 31 years. The mean duration of fever on admission was 4.4 days (range 2-6 days) and the total duration of the illness ranged from 5 to 20 days by the time of death. The serology suggested secondary infection in the series except in case 5 where the infection type was not determined as only an IgM result was available. During the illness the mean lowest platelet count of the cases was 15 × 10^9^/L (range 3-30 × 10^9^/L) and the highest mean haematocrit value was 48% (range 40-54%). These patients developed profound hypotension despite intravenous fluid replacement, tachycardia, pleural effusions and ascites. Only two patients had significant bleeding from mucosae. All of them showed wide spread T wave inversion in 12 lead ECGs and were positive for cardiac troponin T. Three patients who had 2D echocardiograms during the illness showed hypokinetic wall motion and decreased ejection fraction.

### Macroscopic and histopathological findings at autopsy (Table 2 and Figure 2)

**Figure 2:**
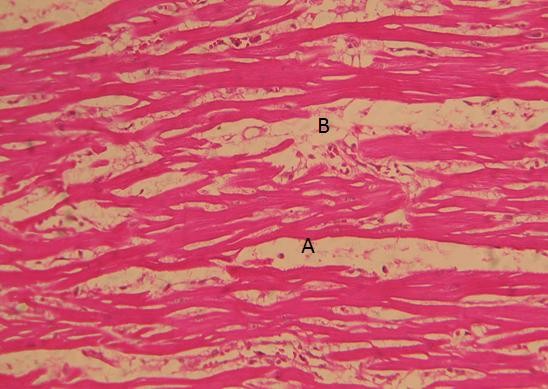
**H&E section of myocardium of a 31-year-old, female showing interstitial oedema(A) and infiltration of inflammatory cells with necrosis of myocardial fibres (B)**.

At autopsy, pleural effusions and ascites were found in the chest and abdominal cavities respectively. There were macroscopic areas of bleeding in muscles and internal organs. Histopathlogically, all 5 cases showed evidence of florid myocarditis. The distinct histological features of the myocardium were: interstitial oedema with inflammatory cell infiltration, necrosis of myocardial fibers and, in one case, evidence of pericarditis. Significant histopathological changes were also seen in the lungs, liver, brain and spleen (Table 2). The distinct pulmonary abnormalities were septal congestion, pulmonary haemorrhage and diffuse alveolar damage. Case 4 showed massive necrosis of the liver. In all cases the pancreas was normal.

## Discussion

The presence of interstitial oedema, infiltration of inflammatory cells and necrosis of myocardial fibers in the myocardium suggested severe myocarditis in all five cases and these features tally with the clinical evidence of cardiac dysfunction they developed during the illness causing profound hypotension and death. In addition to the heart, cellular changes occurred in organs such as lungs and liver together with evidence of bleeding and effusions in to serosal cavities. This particular outbreak carried a high death rate and only in a few cases was DEN-1 shown to be the causative serotype. By performing autopsies and histology, we attempted to confirm the occurrence of myocarditis in dengue infection as a true manifestation. Cardiac dysfunction could also be explained as the result of an indirect mechanism affecting the myocardium. However, without demonstrating viral RNA in the myocardium, the indirect myocardial damage due to cellular immune responses and/or cytokines cannot be excluded. This question was correctly addressed in a recent study where immunohistochemistry was used to demonstrate direct dengue viral infection in the myocardial tissues causing myocarditis [16]. Therefore, the gross histopathological features detected in the current case series (Figure 2) could be due to direct dengue viral infection. The cardiotropism property of the dengue virus and its contribution to morbidity and mortality needs further evaluation. Due to lack of technology in Sri Lanka, the current autopsy study did not proceed to detect dengue viral RNA or antigen in tissues or staining for T-cells (CD3) or macrophages (CD68) to strengthen the histopathological diagnosis of myocarditis.

The characteristics of dengue infection in Sri Lanka have been changing recently in relation to the severity of infection contributing to high morbidity and mortality [2,4]. In 2005, an epidemic showed high incidence of myocarditis, in which the diagnosis of myocarditis was made solely on clinical, ECG and enzyme evidence [4]. Published evidence of cardiac dysfunction in dengue based on clinical manifestations, ECG changes, demonstration of reduced cardiac output by echocardiography and using specific cardiac markers are available in the literature [17-19]. The current study has gone one step further to provide histopathological evidence in support of myocarditis in dengue infection.

There are many viruses recognized as aetiological agents of myocarditis. The pathogenesis of myocartitis caused by these viruses and the related complications are well described in the literature [8-14]. In future, the dengue virus should also qualify as a causative agent of viral myocarditis taking the current evidence into consideration. So far in the literature there are no reports documenting the incidence of myocarditis in dengue infections. The reasons could be due to the rarity of dengue myocarditis or its temporal nature of occurrence or due to under-diagnosis. The experience in Sri Lanka supports its temporal nature of occurrence along with an emergence of new causative dengue serotype. In an epidemic in 2005, DEN-3 was identified as the causative dengue serotype of myocarditis [4], whilst in 2009, DEN-1 was found to be the main serotype responsible for both the island wide outbreak and the regional outbreak at Peradeniya [15]. It is interesting to note that in 2009, DEN-1 emerged as the serotype of importance and caused severe secondary infection among the regional population who had been exposed to DEN-3 in 2005. Thus, viraemia would have been more pronounced in the epidemic in 2009 causing multiple organ dysfunction in addition to excessive fluid leak.

It is well known that the dengue virus has the predilection to infect peripheral blood and vasculature more than vital organs, particularly the myocardium [1,3]. An interesting report published in 2004, used immunohistochemistry and in situ hybridization to localize dengue virus in blood and different human tissues obtained from biopsies and autopsies [20]. They demonstrated the presence of dengue viral antigen in the liver, spleen, lung, kidney and peripheral blood leucocytes, but not in thymus, lymph nodes, thyroid, pancreas, heart, adrenal gland, skeletal muscles, intestine and brain [20]. Even though clinical data are lacking in this paper to understand organ specific clinical manifestations in their patients, it has proved the ability of dengue virus to infect organs. A subsequent study demonstrated direct viral invasion of the myocardium and derangement of calcium storage in the infected cells contributing to damage of myocardial cells [16]. In the current case series, involvement of other organs apart from the heart such as lungs and liver was found. Thus, multiple organ dysfunction and excessive fluid leak as suggested by effusions would have contributed to the final fatal outcome.

## Conclusions

In conclusion, primary cardiac failure due to myocarditis in dengue infection is very often overlooked. The current study presents histopathological evidence to support myocarditis in dengue infection. We emphasise the need to consider the possible existence of myocarditis in severely ill patients with dengue infection in clinical settings. Management guidelines for dengue should stress myocarditis as an important issue.

## Competing interests

The authors declare that they have no competing interests.

## Authors' contributions

SAMK and KGADW conceived the idea. SAMK, KGADW and VGP recorded clinical data. DHE, SKAK, LPGG, ABS did autopsies and histopathological studies. SG did serology. All the authors read and approved the final version of the script.
